# Carbapenem-Resistant *Enterobacter* spp. in Retail Seafood Imported from Southeast Asia to Canada

**DOI:** 10.3201/eid2209.160305

**Published:** 2016-09

**Authors:** Nicol Janecko, Sarah-Lynn Martz, Brent P. Avery, Danielle Daignault, Andrea Desruisseau, David Boyd, Rebecca J. Irwin, Michael R. Mulvey, Richard J. Reid-Smith

**Affiliations:** Public Health Agency of Canada, Guelph, Ontario, Canada (N. Janecko, S.-L. Martz, B.P. Avery, A. Desruisseau, R.J. Irwin, R.J. Reid-Smith);; University of Guelph, Guelph (N. Janecko, S.-L. Martz, R.J. Reid-Smith);; Public Health Agency of Canada, St. Hyacinthe, Quebec, Canada (D. Daignault);; Public Health Agency of Canada, Winnipeg, Manitoba, Canada (D. Boyd, M.R. Mulvey)

**Keywords:** carbapenem resistant, resistance, carbapenemase-producing, gram-negative, Enterobacteriaceae, Enterobacter spp., Enterobacter cloacae, Enterobacter aerogenes, carbapenemase-producing, bacteria, Canada, Southeast Asia, Bangladesh, Vietnam, imported, shrimp, clams, seafood, food chain, antimicrobial resistance

**To the Editor:** Carbapenems, antimicrobial drugs of last resort, are recommended only for severe community- and healthcare-associated multidrug-resistant bacterial infections. In Canada, carbapenem-resistant infection rates in hospitals remained low (<0.25 cases/1,000 patient admissions) over 5 years’ (2009–2014) surveillance (*1*). Carbapenemase-producing bacteria have rarely been detected in the food chain in industrialized countries. However, carbapenemase genes were detected in bacteria isolated from produce in Switzerland (*2*) and seafood in Canada (*3*); implicated food items originated from Southeast Asia. We conducted targeted sampling to assess, using selective media, the occurrence of carbapenem-resistant *Enterobacteriaceae* in imported seafood products sold in Canada.

For testing, we selected 1,328 retail seafood samples: 928 were imported fresh and frozen raw shrimp collected during 2011–2015 by CIPARS (the Canadian Integrated Program for Antimicrobial Resistance Surveillance), and 400 comprised an assortment of imported niche-market fresh and frozen raw seafood collected specifically for this study during January–April 2015. Product information and origin country were recorded for each sample. We used chromID CARBA agar (bioMérieux, St. Laurent, QC, Canada) to select putative colonies. To determine carbapenemase production on nonsusceptible (zone of inhibition <25 mm) isolates, we used disk diffusion susceptibility to ertapenem and meropenem (10 μg each) and the Carba NP test as previously described (*4*). Isolates were identified to species using matrix-assisted laser desorption/ionization time-of-flight mass spectrometry (Bruker Daltonics Ltd, Milton, ON, Canada) and tested for susceptibility using the Sensititre Complete Automated System with the Sensititre NARMS Gram Negative Plate (CMV3AGNF) (Trek Diagnostic Systems, Oakwood Village, OH, USA). We used single and multiplex PCR to screen isolates for the major carbapenemase-conferring (*bla*_NDM_, *bla*_KPC_, *bla*_IMP_, *bla*_VIM_, *bla*_GES_, *bla*_OXA-48–like_, *bla*_NMC_) and β-lactamase–conferring (*bla*_SHV_, *bla*_TEM_, *bla*_CTX-M_, *bla*_OXA-1_, *bla*_CMY-2_) genes (*5*). We performed pulsed-field gel electrophoresis (PFGE) and whole-genome sequencing (Illumina Inc., San Diego, CA, USA) on isolates requiring further comparative testing (*6*). In silico multilocus sequence typing and replicon typing were conducted using the assembled sequence data (SPAdes 3.5.0 [St. Petersburg genome assembler], http://spades.bioinf.spbau.ru/release3.5.0/manual.html) and services of the Center for Genomic Epidemiology (http://www.genomicepidemiology.org). The transferability of resistance genes was determined by transformation experiments using eletrocompetent *Escherichia coli* DH10B cells.

Using selective media methodology, we detected carbapenem-resistant *Enterobacteriaceae* in 8 (0.6% [95% CI 0.26–1.18]) of the 1,328 seafood samples; all 8 were from Southeast Asia (Table). Of the 928 shrimp samples collected as part of CIPARS sampling, 2 (0.2% [95% CI 0.03–0.78]) imported from Vietnam contained *Enterobacter cloacae* harboring *bla*_IMI −1_, and 1 (0.1% [95% CI 0.003–0.599]) from Bangladesh contained *E. aerogenes* harboring *bla*_IMI-2_. Of 101 mollusk samples, 3 (3.0% [95% CI 0.62–8.44]) clam samples imported from Vietnam contained *E. cloacae* harboring *bla*_IMI −1_, and 2 (2.0% [95% CI 0.24–6.97]) clam samples from Vietnam contained *E. cloacae* harboring *bla*_NDM-1_, *bla*_TEM_, and *bla*_OXA-1_. All isolates with carbapenemase genes were phenotypically resistant to ampicillin, cefoxitin, and amoxicillin/clavulanic acid; some were multiclass-resistant (Table).

**Table:** Carbapenem-resistant *Enterobacter* species detected in retail seafood products imported from Southeast Asia to Canada*

Sample type, resistant species	No. (%) samples [95% CI]	Origin of seafood	Gene	Antibiogram profile	ST†
Shrimp, n = 928					
* E. cloacae*	2 (0.2) [0.03–0.78]	Vietnam	*bla*_IMI-1_	AMC-AMP-(AZM)-FOX‡	ST411; ST412
* E. aerogenes*	1 (0.1) [0.003–0.599]	Bangladesh	*bla*_IMI-2_	AMC-AMP-FOX	NA
Bivalve mollusks, n = 101					
* E. cloacae*	2 (2.0) [0.24–6.97]	Vietnam, clam	*bla*_NDM-1_, *bla*_TEM_, *bla*_OXA-1_	AMC-AMP-FOX-TIO-CRO-CHL-CIP-GEN-STR-FIS-TET-TMP/SXT	ST479
* E. cloacae*	3 (3.0) [0.62–8.44]	Vietnam, clam	*bla*_IMI-1_	AMC-AMP-(AZM)-FOX§	ST477; ST478; ST373
Cephalopods, n = 240	0 [0.00–1.53]	NA	NA	NA	NA
Miscellaneous, n = 59	0 [0.00–6.06]	NA	NA	NA	NA

*AMC, amoxicillin/clavulanic acid; AMP, ampicillin; AZM, azithromycin; CHL, chloramphenicol; CIP, ciprofloxacin; FIS, sulfisoxazole; CRO, ceftriaxone; FOX, cefoxitin; GEN, gentamicin; NA, not applicable (no scheme found); ST, sequence type; STR, streptomycin; TET, tetracycline; TIO, ceftiofur; TMP/SXT, trimethoprim/sulfamethoxazole. †Determined by multilocus sequence typing. ‡ST412 resistant to AZM. §ST477 and ST373 resistant to AZM.

Isolates harboring *bla*_IMI-1_ genes contained no plasmid DNA. However, using electroporation into *E. coli*, we showed that the *bla*_IMI-2_ gene was plasmid-mediated; the plasmid contained the IncFII(Yp) replicon. The *bla*_NDM-1_ genes were nontransformable into *E. coli*, although the 2 isolates contained IncHI2, IncFIB, and IncFII replicons. The location of the *bla*_NDM-1_ gene may therefore be chromosomal or plasmidic. Six different sequence types (STs) of *E. cloacae* were shown by multilocus sequence typing. PFGE results showed that the 2 *E. cloacae* ST479 isolates were indistinguishable, whereas the other isolates were distinct. The *E. cloacae* ST479 isolates harbored *bla*_NDM-1_, *bla*_OXA-1_, and *bla*_TEM_; were phenotypically resistant to 12 tested antimicrobials drugs; and were from clam samples collected at different retail outlets on different dates. Comparison of ST373 fingerprints with the National Microbiology Laboratory PFGE database containing >170 *E. cloacae* of human origin showed that a human-sourced *E. cloacae* ST373 isolate harboring *bla*_IMI-1_ shared >75% similarity with a clam-sourced *E. cloacae* isolate. In addition to the carbapenem-resistant *Enterobacteriaceae* findings described here, our findings also show that 1 sample, from a black tiger shrimp (*Penaeus monodon*) originating from India, contained a non-O1, non-O139 *Vibrio cholerae* with a novel class A carbapenemase gene named *bla*_VCC-1_ (GenBank accession no. KT818596); this isolate has been described elsewhere (*6*).

Seafood, such as shrimp and clams, are raised in aquatic environments with a known potential for water-source contamination (*7*,*8*). We found multiple retail seafood samples containing *Enterobacter* spp. harboring *bla*_NDM-1_ and *bla*_IMI-type_ genes. This finding suggests that, for humans, the source of carbapenemase-producing *Enterobacter* spp. may not be limited to exposure during travel; contaminated food products may also be a source of exposure (*9*). The identification, in imported clams, of *E. cloacae* with the same ST and similar DNA fingerprint pattern as an isolate from a human raises concerns of a possible association; however, more work is required before a linkage and direction of transfer can be inferred. Our findings highlight the need for antimicrobial resistance surveillance systems to consider the use of selective media methodology to increase sensitivity for the detection of rare or emerging resistance genes.
